# *In vivo* biocompatibility and immunogenicity of metal–phenolic gelation†
†Electronic supplementary information (ESI) available. See DOI: 10.1039/c9sc03325d
‡
‡Code used for the Raman spectroscopy studies in this work is available online at https://github.com/conor-horgan/TiTannic-Gels.


**DOI:** 10.1039/c9sc03325d

**Published:** 2019-09-25

**Authors:** Mattias Björnmalm, Lok Man Wong, Jonathan P. Wojciechowski, Jelle Penders, Conor C. Horgan, Marsilea A. Booth, Nicholas G. Martin, Susanne Sattler, Molly M. Stevens

**Affiliations:** a Department of Materials , Department of Bioengineering , Institute of Biomedical Engineering , Imperial College London , London SW7 2AZ , UK . Email: m.stevens@imperial.ac.uk; b National Heart and Lung Institute , Imperial College London , London W12 0NN , UK . Email: s.sattler@imperial.ac.uk; c Trace Element Laboratory , North West London Pathology , Charing Cross Hospital , London W6 8RF , UK

## Abstract

*In vivo* forming hydrogels are of interest for diverse biomedical applications due to their ease-of-use and minimal invasiveness and therefore high translational potential. Supramolecular hydrogels that can be assembled using metal–phenolic coordination of naturally occurring polyphenols and group IV metal ions (*e.g.* Ti^IV^ or Zr^IV^) provide a versatile and robust platform for engineering such materials. However, the *in situ* formation and *in vivo* response to this new class of materials has not yet been reported. Here, we demonstrate that metal–phenolic supramolecular gelation occurs successfully *in vivo* and we investigate the host response to the material over 14 weeks. The Ti^IV^–tannic acid materials form stable gels that are well-tolerated following subcutaneous injection. Histology reveals a mild foreign body reaction, and titanium biodistribution studies show low accumulation in distal tissues. Compared to poloxamer-based hydrogels (commonly used for *in vivo* gelation), Ti^IV^–tannic acid materials show a substantially improved *in vitro* drug release profile for the corticosteroid dexamethasone (from <1 day to >10 days). These results provide essential *in vivo* characterization for this new class of metal–phenolic hydrogels, and highlight their potential suitability for biomedical applications in areas such as drug delivery and regenerative medicine.

## Abstract 2

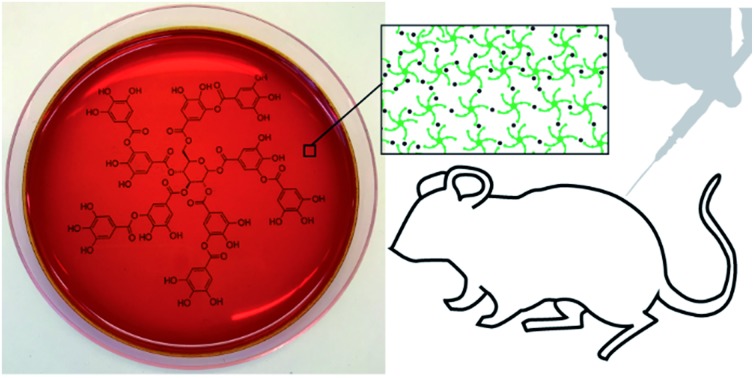
Coordination-driven supramolecular *in vivo* assembly of metal–phenolic hydrogels.

## Introduction

Supramolecular biomaterials and hydrogels have gained great interest in biomedical engineering for applications such as drug delivery, tissue engineering, regenerative medicine and immunology.^1^,^2^ Supramolecular hydrogels are assembled by interlinking small molecules in a noncovalent fashion, for example through metal coordination.^3^ One such example is the coordination between metals and phenolic compounds, which has previously been used to engineer conformal coatings and thin films,^4^–^6^ as well as to stabilize hydrogels prepared using pre-synthesized macromolecular building blocks.^7^–^9^ Metal–phenolic materials are of increasing interest across the chemical sciences for the preparation of functional materials in diverse application areas such as adhesives, self-cleaning surfaces, separation, catalysis, crystallization, sensing, drug delivery and imaging.^10^–^12^ Increasing our understanding of the biological interactions of metal–phenolic materials is paramount for continued research in this emerging area of materials science, as it is a prerequisite for understanding their toxicology, environmental impact and potential in biomedicine.

Recently, metal–phenolic supramolecular gelation was demonstrated through the direct gelation between naturally occurring, unmodified polyphenols and group IV metal ions (Ti^IV^ or Zr^IV^).^13^ This coordination-driven supramolecular assembly can be initiated by simple mixing at ambient conditions in various solvents over large concentration ranges and metal–ligand stoichiometries.^13^ The result is robust and adaptive gels, which can be used for controlling concurrent assembly processes (*e.g.*, crystallization of metal–organic frameworks or pharmaceuticals), or for *in situ* co-gelation of diverse dopants.^13^,^14^ While the *in vitro* cytotoxicity of this new class of materials based on metal–phenolic supramolecular gelation has been studied and observed to be negligible,^13^*in vivo* studies are yet to be reported.

*In vivo* gelation, where hydrogels form spontaneously *in situ* under physiological conditions (*e.g.*, at the site of injection), is of broad interest for biomedical applications due to its minimal invasiveness and high translational potential.^15^,^16^ Examples include thermosensitive poloxamer-based gels used for antibiotic delivery to the inner ear,^17^ hydrophobically modified poly(ethylene glycol) gels for ophthalmic applications,^18^ and cell sheet transplantation using thermosensitive gelatin-based hydrogels.^19^ Many biomedical applications also use gel systems such as calcium–alginate gels which are based on ionic crosslinking of the biomolecule alginate with Ca^2+^. Calcium–alginate gels typically form rapidly upon mixing.^20^,^21^ While strategies to modulate the gelation kinetics exist (*e.g.*, using calcium-releasing liposomes to trigger gelation^22^ or phosphate salts to slow gelation time^23^), pre-formed alginate gels such as implantable beads remain the most commonly used in both pre-clinical and clinical research.^24^–^28^ In contrast, gelation times for recently introduced^13^ metal–phenolic gels—prepared through coordination of the biomolecule tannic acid (Fig. S1 in ESI†) with Ti^IV^—can be readily tuned from less than 1 minute to more than 1 day in a robust gelation process that is insensitive to a large range of conditions. We thus hypothesize that these Ti^IV^–tannic acid gel systems (“TiTannic gels”) are suitable candidates for *in vivo* gelation and future biomedical applications.

**Fig. 1:**
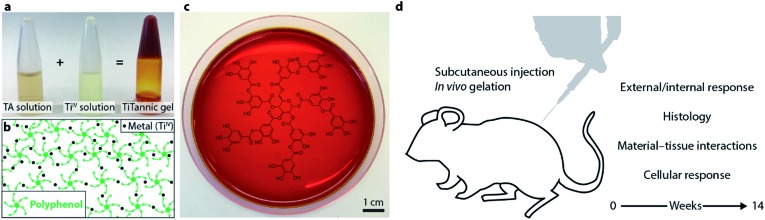
*In vivo* metal–phenolic supramolecular gelation. (a) Photographs showing that upon mixing of TA solution with a Ti^IV^ solution, a TiTannic hydrogel is formed. (b) Schematic illustrating that the gel is stabilized by metal–phenolic coordination between the polyphenol and the metal ion. (c) Photograph of a prepared gel (5 mm in thickness) in a petri dish, showing the transparency of the gel. Underneath the petri dish is the chemical structure of tannic acid (Fig. S1†) printed on paper. (d) Overview of *in vivo* biocompatibility and immunogenicity study.

Here, we demonstrate that metal–phenolic supramolecular gelation occurs *in vivo* and investigate the host response to the material over 14 weeks (Fig. 1). Liquid precursors (tannic acid solution and Ti^IV^ solution) were prepared and filter-sterilized. The composition was tailored to achieve a gelation time of around 15 minutes, which was deemed suitable for allowing careful injection. Prior to animal studies, the gel system was characterized using electron microscopy and Raman spectroscopy, and permeability and porosity were assessed using a glucose permeability assay and particle tracking analysis, respectively. For the animal studies, the two sterile precursors were mixed immediately prior to subcutaneous injection in the flanks of immunocompetent mice. At specified time points during the 14 week period, external and internal photographs of the injection sites were taken and histological sections were prepared. Additionally, tissue samples were collected for titanium biodistribution studies using mass spectroscopy. Titanium remained largely at basal levels for most studied tissues and time points, indicating low to negligible titanium accumulation. Finally, drug loading and elution studies were performed *in vitro* using the corticosteroid dexamethasone, and drug elution from the TiTannic gels was observed over a period of >10 days, which can be compared to <1 day for the Pluronic F127 hydrogels prepared as controls.

Taken together, these results demonstrate that metal–phenolic supramolecular gelation occurs *in vivo*, and provide essential *in vivo* characterization for this emerging class of materials. The observed *in vivo* responses are largely comparable with those previously reported for conventional calcium–alginate gels.^24^–^28^ Additional benefits of the TiTannic system, including readily tuneable gelation time, robustness and adaptability, and the easy, low-cost, off-the-shelf, and scalable preparation process, make this new class of material of significant interest for diverse biomedical applications.

## Results and discussion

### Electron microscopy, Raman spectroscopy and rheology of the hydrogels

**Fig. 2:**
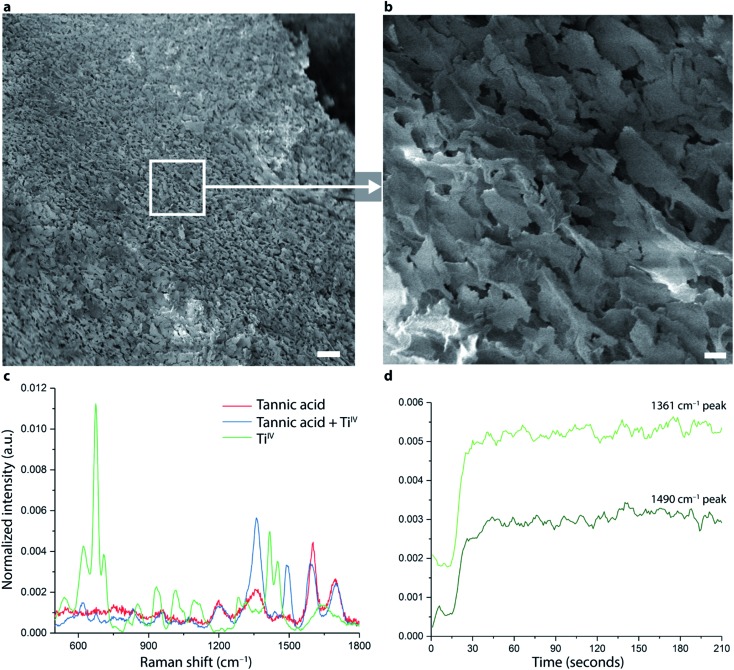
SEM and Raman spectroscopy of TiTannic gels. (a and b) SEM images of internal microscopic structure of a TiTannic gel following freeze-fracture in liquid nitrogen, freeze-substitution in acetone, and lyophilization. Panel (b) is an enlargement of the white square in panel a. Note that these images are not of hydrated gels. Scale bars are 30 μm and 2 μm for (a and b), respectively. (c and d) Raman spectroscopy during gelation using an *in situ* Raman probe and spectral analysis. (c) Raman spectra of constituent parts and the mixture. (d) Peaks at 1361 cm^–1^ and 1490 cm^–1^ appear within 30 seconds after mixing and stabilize within 1 minute. Time = 0 is when TA solution and Ti^IV^ solution were mixed.

Metal–phenolic supramolecular gelation occurs spontaneously upon mixing of polyphenols (*e.g.* tannic acid, TA) with group IV metal ions (*e.g.* Ti^IV^). This class of material was recently introduced and has been characterized with rheological methods, optical microscopy, UV-vis spectroscopy, X-ray photoelectron spectroscopy and X-ray diffraction among other techniques.^13^,^14^ To further explore the nanostructure of the gel, we performed a freeze-fracture procedure using liquid nitrogen, followed by freeze-substitution in acetone and lyophilization before scanning electron microscopy (SEM) imaging (Fig. 2). The results show a structure with porosity on the micrometer and nanometer length scales (Fig. 2a and b). It should be noted, however, that whilst the freeze-substitution and lyophilization process may affect the pore structure compared to the pristine hydrated material, the technique can still provide valuable insight into pore structure and levels of hierarchy.^29^

Raman spectroscopy has been extensively used to investigate materials prepared using coordination-driven assembly and coordination compounds.^30^,^31^ We applied a Raman spectroscopy probe with continuous spectral acquisition (with 1 second integration time) to obtain Raman spectra *in situ* during gelation (Fig. S2†), the results are shown in Fig. 2c and d. When comparing Raman spectra of the individual components (TA solution and Ti^IV^ solution) with that of the formed gel (TA + Ti^IV^), two enhanced peaks, at 1361 cm^–1^ and at 1490 cm^–1^, are observed (Fig. 2c). These peaks increased in intensity during the first 30 seconds after mixing, and then remained largely stable during the whole experiment (1 hour). The observed changes correspond to previously reported Raman shifts for metal–phenolic systems,^32^ which were assigned to skeletal modes of the substituted benzene rings and stretching of carboxylate groups of the phenolic compounds interacting with the metal ligands. Metal–phenolic networks can also be redox-active as has been recently reported by an electrochemical study,^33^ and future studies combining Raman spectroscopy with electrochemical analysis may be of interest to elucidate these molecular interactions further. The TiTannic hydrogels were measured using rheology (Fig. S3†), displaying a storage modulus (*G*′ = 12.6 kPa) significantly larger than the loss modulus (*G*′′ = 0.595 kPa). An amplitude sweep was conducted to determine the linear viscoelastic region and crossover points of the hydrogels, whereas the frequency sweep showed frequency independent behavior of the hydrogels. The time sweep showed gelation occurred within 15 minutes, with the hydrogels ageing slowly over ∼15 hours. Gelation, as determined using vial inversion (Fig. 1a), occurred within 15 minutes and is consistent with the gelation kinetics measured by rheology (Fig. S3†). At earlier time points (*e.g.* 30 seconds after mixing) the mixture is still liquid and will not remain stationary if the vial is inverted. The Raman probe provides spectral information of a local volume (∼1 mm from the probe tip) and these data therefore suggest that local coordination occurs rapidly (within 1 minute), which is in agreement with previously reported metal–phenolic nanoscale films and coatings.^4^ Shorter range (nanoscale) interactions in the material may thus be completed within seconds, while longer-range interactions needed to form the stable gel were observed to take around 15 minutes for the composition used here. It is noted that for other compositions, gelation time can vary drastically, from <1 minute to >1 day.^13^

### Hydrogel porosity and permeability

**Fig. 3:**
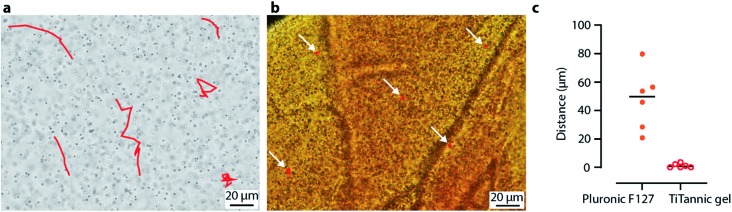
TiTannic gels have sub-micron pores. Particle tracking analysis of polystyrene particles (1 μm in diameter) in Pluronic F127 gel (a) and TiTannic gel (b). Red lines correspond to the movement of a single particle during 1 minute. White arrows indicate red trace lines in (b); these particles remained largely stationary (<5 μm distance travelled). (c) Displacement distance measured using particle tracking analysis. Dots are individual data points and the lines indicate the median.

To further investigate the porosity of the hydrated material, particle tracking analysis was used (Fig. 3). In this method, microparticles are dispersed in the gel (through co-gelation) and their movement is tracked over time using optical microscopy. This information can then be used to explore the porosity and functional tortuosity of a material.^34^,^35^ We compared the movement of embedded 1 μm polystyrene particles in Pluronic F127 hydrogels and in TiTannic gels at 37 °C. Pluronic F127 gelation is a well-established gelation system (in this study acting as comparative control samples) that is thermoresponsive and where the gelation occurs through a micellar-packing mechanism.^36^,^37^ In the Pluronic F127 gel, a particle movement of around 50 μm (median displacement distance) per minute was observed (Fig. 3c). That particles can easily move around is expected as the micellar-packing structure can accommodate substantial flexibility and movement inside the gel.^36^,^37^ In contrast, for TiTannic gels the median displacement distance per minute was 10-fold lower (<5 μm; Fig. 3c). The largely stationary behavior of particles embedded in the TiTannic hydrogels indicates that the effective hydrated pore size is smaller than the particle diameter (*i.e.*, 1 μm). This difference in effective pore size between the two different gel systems may be explained by the differences in chemistry and assembly mechanism: whereas Pluronic F127 gels are composed of macromolecules (poloxamers, >10 kDa) that form micelles,^38^ TiTannic gels are instead assembled through molecular coordination between metal ions and low molecular weight polyphenol (TA, 1.7 kDa). For future studies it may be of interest to expand on these results using nanoparticles and super-resolution microscopy, as has been recently done for other types of gel materials.^34^,^35^

**Fig. 4:**
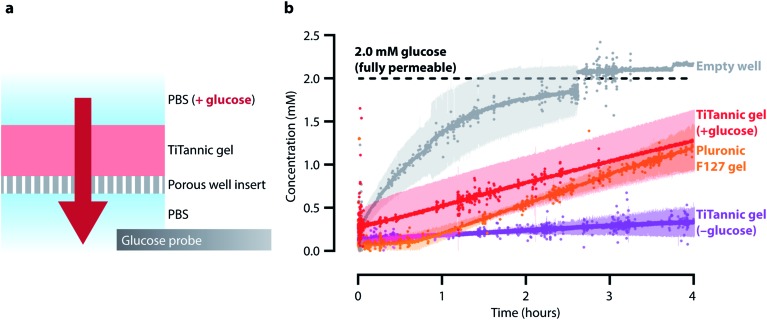
TiTannic gels are permeable to glucose. (a) Schematic of the glucose permeability assay. (b) Measured glucose permeability for a TiTannic gel and a Pluronic F127 gel. An empty well (no gel added) was used as a control, representing free diffusion of glucose through the well insert. The dotted black line corresponds to 2.0 mM glucose concentration which is the equilibrated glucose concentration on both sides of the well insert membrane (*i.e.* full permeability). TiTannic gels without any glucose added were used as controls to confirm that the gel itself did not induce any substantial signal with the glucose probe. Dots represent average values of duplicates and the shaded areas represent the standard deviation.

After having established that microparticles remain largely stationary in TiTannic gels, we investigated the diffusion rate of a small molecule: glucose (0.18 kDa). Glucose-permeable hydrogels are of interest clinically, as these types of gels are being explored to encapsulate insulin-producing cells for the treatment of diabetes.^23^,^24^,^28^ In the present investigation, we developed a glucose permeability assay (Fig. 4) to compare glucose permeability of the TiTannic gel system to Pluronic F127 gels, an *in vivo* gelation system which has previously been explored for the treatment of diabetes.^39^ In the glucose permeability assay, the gel was cast in a porous well insert (membrane with 0.4 μm pores) and PBS was added both underneath and above the gel following gelation (Fig. 4a). Concentrated glucose was then added above the gel, while a glucose probe was situated underneath the gel (Fig. S4†). In the empty well control (*i.e.* free diffusion), the glucose concentration was measured as equilibrated within the first three hours, and for the TiTannic control sample (no glucose added) the signal remained low, as expected (Fig. 4b). For both Pluronic F127 and TiTannic gels, retarded diffusion relative to the empty well (free diffusion) was observed. While there is a large overlap in the observed glucose concentration increase during the first few hours (Fig. 4b), Pluronic F127 gels appeared to stabilize at longer time points (>10 hours) at a higher concentration than the TiTannic gels (Fig. S5†). Pluronic F127 gels are known to dissolve over time which may contribute to this difference.^36^ Polyphenols are known to interact with carbohydrates,^40^ and this may contribute to the observed lower glucose level detected. Nevertheless, the results demonstrate that Pluronic F127 gels and TiTannic gels are both permeable to glucose.

### TiTannic gels are stable and well tolerated after subcutaneous injection in mice

**Fig. 5:**
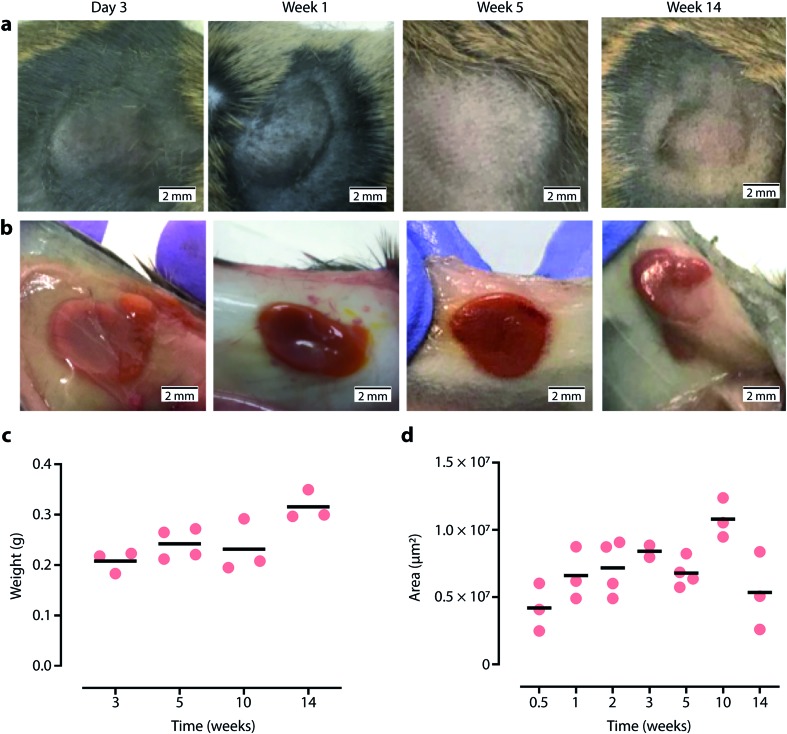
TiTannic gels are stable and well tolerated after subcutaneous injection in mice. Macroscopic appearance of the TiTannic gel over time as observed (a) externally after hair removal, and (b) internally. (c) Gel weight over time measured after excision including overlaying skin area. (d) Gel size over time as measured by cross-sectional area in H&E histology section. Data points in (c and d) indicate a sample from an individual animal (*N* = 2–4), and horizontal lines indicate mean value.

For initial assessment of TiTannic gel tolerability, an individual mouse was injected subcutaneously on the right flank with 50 μL of gel precursors. Precursors were always mixed immediately before injection and had a pH of around 7 (see Section 1 in ESI†). The initial assessment using an individual mouse was followed by a second mouse injected subcutaneously with 100 μL of gel precursors. The gels formed a solid disc-shaped mass within ∼15 minutes, which was palpable underneath the skin. As no systemic or local adverse effects were observed for these first mice, a subsequent group of mice was subcutaneously injected with 100 μL of TiTannic gel precursor and culled at selected time points up to week 14 post-injection. The injection site was investigated and resected for histological assessment. Distant tissue samples were collected (including brain, heart, kidney, liver, lung and spleen) to measure systemic levels of titanium. Gross macroscopic assessment at termination showed negligible changes in external as well as internal appearance of the injection site (Fig. 5a and b). The small variation in gel mass (∼0.2–0.3 g) between animals is likely due to variation introduced during injection and processing for histology (Fig. 5c). No substantial swelling or shrinkage of the gels was observed, and gel sizes remained largely constant over the duration of the experiment (Fig. 5d). These results show that TiTannic gels are stable *in vivo*, which is in agreement with the *in vitro* disassembly studies (Fig. S6 and S7†).

### TiTannic gels elicit a mild but persistent foreign body reaction

**Fig. 6:**
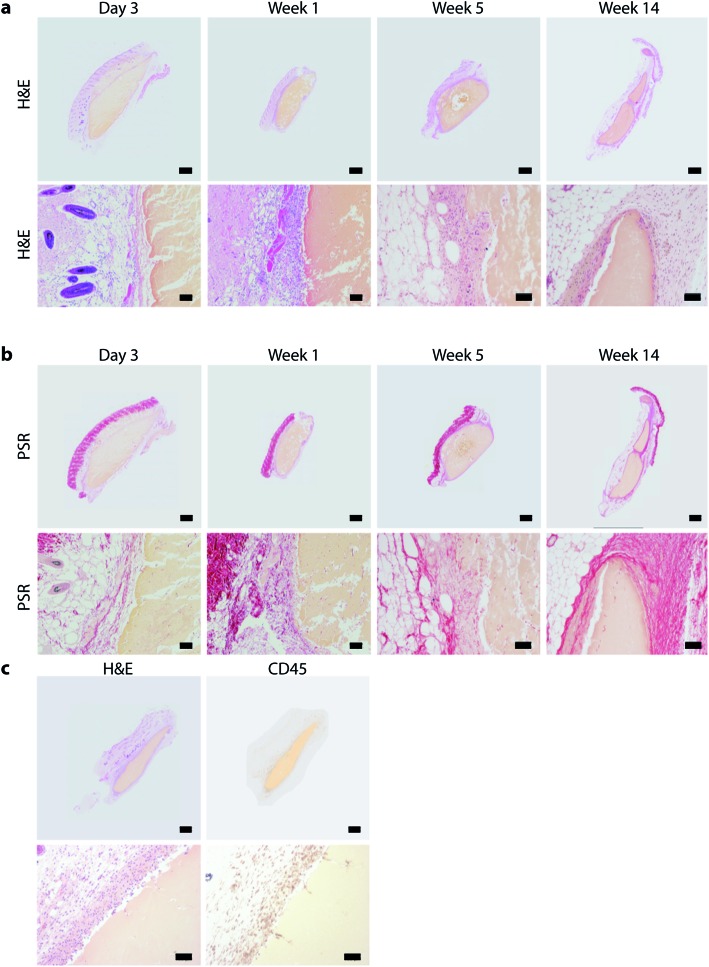
Histology of TiTannic gel underneath the skin. Representative (a) haematoxylin and eosin (H&E) and (b) Picrosirius red (PSR) histology staining of paraffin embedded TiTannic gel cross sections over time (H&E; blue nuclei, pink cytoplasm, yellow TiTannic gel. PSR; red collagen fibers, yellow TiTannic gel). (c) Example of H&E section compared to anti-CD45 immunohistochemistry staining (brown). Scale bars are 1000 μm (top rows, full gel images) and 100 μm (bottom rows, magnified views).

After excision, gels were processed for histology and stained with haematoxylin & eosin (H&E) to assess immune cell accumulation around the gel and Picrosirius red to visualize collagen deposition and the formation of a fibrotic capsule. The entire gel underneath the skin and embedded in subcutaneous tissue is shown in Fig. 6 and S12.† Negligible cell infiltration or collagen deposition was observed after 3 days but increased by week 1 showing a clear disorganized mononuclear infiltrate scattered with individual collagen fibers around the gel. This inflammatory reaction was then observed to follow the common course of a foreign body reaction: mononuclear cells organized into layers of epithelioid histiocytes gave the foreign body capsule a homogenous appearance. Foreign body giant cells also started to appear at the border of the gel. From week 2 onwards, areas of increasing overlap between the gels and surrounding tissue were observed (Fig. 6). A thin but homogenously organized fibrotic capsule was visible at later time points. As expected, most cells surrounding the gel stained positive for CD45, a pan-leukocyte marker (Fig. 6c).

**Fig. 7:**
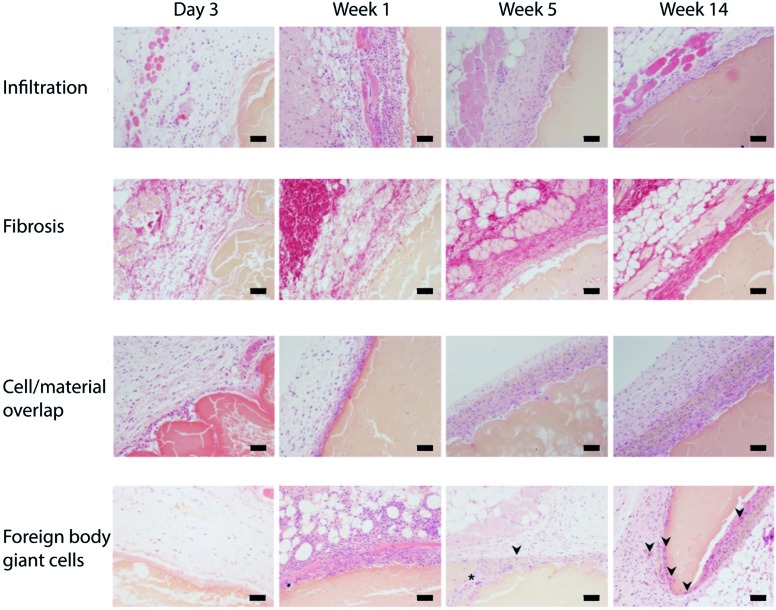
TiTannic gels elicit a mild but persistent foreign body reaction. Representative micrographs of infiltration layer, fibrosis layer, cell/material overlap layer thickness and number of foreign body giant cells (FBGC) over time. Infiltration, cell/material overlap thickness and number of FBGC stained using haematoxylin and eosin, and fibrosis layer thickness stained using Picrosirius red. In the FBGC row, asterisks indicate macrophage-derived FBGC and arrow indicates Langerhans cell-derived FBGC. All histology staining was performed in paraffin-embedded TiTannic gel cross-sections. Scale bars are 100 μm.

**Fig. 8:**
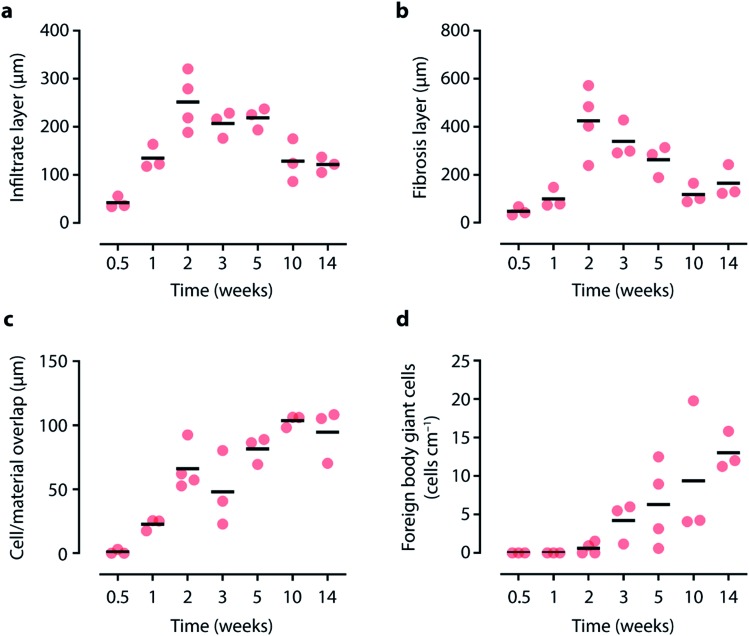
Quantifying tissue–material interactions. Measurements based on representative micrographs of (a) infiltration layer, (b) fibrosis layer, (c) cell/material overlap layer thickness and (d) number of foreign body giant cells over time. See Fig. 7 for example histology micrographs. Data points indicate tissues from an individual animal (*N* = 3–4), and horizontal lines indicate mean value.

A quantitative time course of the development of the foreign body reaction and representative histology images are shown in Fig. 7 and S11,† and quantification is presented in Fig. 8. The thicknesses of inflammatory infiltrate (Fig. 8a) and fibrotic layer (Fig. 8b) followed a similar curve with an early increase and a gradual decline over time. As expected, fibrosis developed with a brief delay as a result of immune cell infiltration. On the other hand, the thickness of the overlap layer between gel and cellular infiltrate increased over time (Fig. 8c), as did the number of foreign body cells (Fig. 8d and S13†).

The above indicates that TiTannic gels are largely stable *in vivo*, with limited degradation observed during the experiment, which is in agreement with the *in vitro* results (Fig. S6 and S7†). However, interestingly, increasing interactions with surrounding tissue and cells over time was observed. For example, it appears that small pieces of gel broke off the bulk material and were being taken up by surrounding cells, which may eventually lead to disassembly and degradation of the gel over extended periods of time. The disassembly of TiTannic gels can occur through pH-induced changes in the molecular interactions of the materials, and/or through the presence of competing ligands (Fig. S6†). For metal–phenolic materials in general, the disassembly behavior can be tuned by tailoring the composition (*e.g.* using blends of phenolic ligands).^6^,^10^,^33^ An interesting research direction is, therefore, the engineering of metal–phenolic gels with disassembly profiles tailored to specific applications, such as rapid biodegradation. Metal–phenolic hydrogels can also be prepared by replacing titanium with zirconium as the coordinating metal.^13^ Exploring these zirconium-based hydrogels would be of interest for future studies as zirconium-containing materials have shown improved performance (even compared to well-performing titanium-containing materials) in some biomedical applications.^41^

### Titanium accumulation is low in distal tissues

**Fig. 9:**
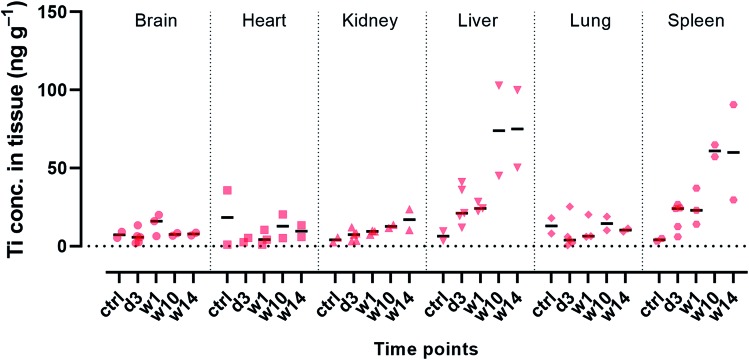
Biodistribution studies of titanium reveal low levels in most distal tissues, with some accumulation observed in liver and spleen. Quantification of titanium levels was performed using mass spectroscopy following tissue digestion. Control samples (ctrl) are from animals that were not exposed to TiTannic gels. Tissues from animals exposed to TiTannic gels were collected 3 days (d3) and 1, 10 and 14 weeks (w1, w10, w14, respectively) following subcutaneous injection of TiTannic gel. Data points indicate tissues from an individual animal (*N* = 2–5), and horizontal lines indicate median value.

Coordination-driven assembly strategies are based around non-covalent interactions, which are dynamic with a stability that depends on the local environment and the time scale.^2^,^3^ For example, TiTannic hydrogels can disassemble rapidly (within minutes to hours) if exposed to extreme pH or to competing ligands (Fig. S6†), but remain stable for months when immersed in sterile cell culture media and PBS (Fig. S7†). While no large changes in gel size or mass were observed during our *in vivo* studies (Fig. 5), small amounts of material leakage from the TiTannic gels could still occur. Tannic acid is a naturally abundant polyphenol (found in many plants, fruits and vegetables) and is being investigated for its intrinsic therapeutic effects and its ability to enhance the delivery of drugs.^42^,^43^ The other component, titanium, is a metal commonly used in the design of implants (*e.g.*, for dental implants and joint replacement).^44^–^46^ Detection of increased tissue levels of titanium is of interest clinically to aid in the assessment of implant status and prognosis, where increased levels of titanium may indicate increased implant wear.^46^ In the current study, we used mass spectroscopy to evaluate titanium levels at distal tissues to the injection site (Fig. 9).

For brain, heart, kidney and lung tissues no large differences were observed in titanium concentration for tissue samples from mice which had been subcutaneously injected with TiTannic gels compared to tissue samples from mice which had not been exposed to TiTannic gels. The titanium concentration in these tissues was below 50 ng Ti per g of tissue (Fig. 9). This is in agreement with previously reported results for unexposed rodents.^47^ A background level is expected as titanium is abundant in our environment, especially in the form of TiO_2_ (*e.g.* in pigments, sunscreens, toothpaste, paints) with median daily adult intake in the UK being approximately 2.5 mg.^48^,^49^ For patients with titanium-containing implants, soft tissue Ti concentrations of around 1 × 10^6^ ng Ti per g tissue have been reported directly adjacent to the implant, and concentrations around 6500 ng Ti per g for tissues collected 3 cm from the implant.^45^ In the present study, increased Ti concentrations were observed for liver and spleen tissues at the longer time points (Fig. 9), with concentrations of around 50–100 ng Ti per g of tissue. The overall low titanium concentrations observed is in agreement with the low gel disassembly observed both *in vitro* and *in vivo* (Fig. 5, S6 and S7†).

Titanium concentration in blood and urine samples collected from the animals were also determined using mass spectroscopy (Fig. S8†). At earlier time points (3 days and 1 week) a 5–10-fold increase in Ti concentration was observed for the blood samples obtained from animals subcutaneously injected with TiTannic gel compared to blood obtained from unexposed animals: from around 1 μg Ti per L of blood to 5–10 μg per L. Basal concentrations on the order of 1 μg Ti per L of blood is routinely reported for unexposed rodents and patients./50 Ti concentration in urine samples was low (close to limit of detection) for all samples studied (Fig. S8†), as expected as the metal is not readily excreted into the urine.^46^ Taken together with the observed Ti accumulation in the liver (Fig. 9), this suggests that a possible excretion mechanism may instead be through the feces.^51^ Feces were not collected and analyzed in the current study, but may be of interest for future studies.

### TiTannic gels exhibit more sustained drug release compared to Pluronic F127 gels

**Fig. 10:**
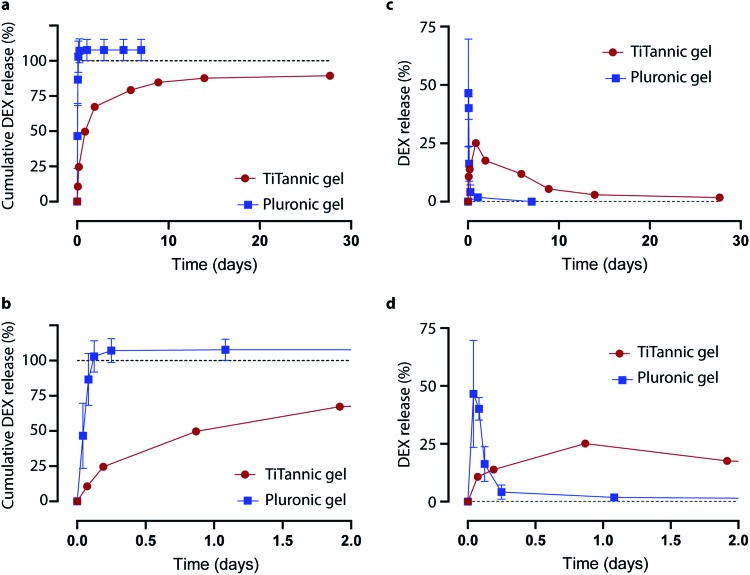
TiTannic gels exhibit more sustained drug release than Pluronic F127 gels. (a) Cumulative dexamethasone (DEX) release from hydrogels. 100% release (all drug released) is indicated with a dotted line. (b) Cumulative drug release over the first two days (same data as in panel a, but *x*-axis 2 days instead of 30 days). Drug release per time point (*i.e.*, non-cumulative) over 30 days (c) and the first two days (d). The data points indicate the average of triplicates with error bars indicating standard deviation (error bars are inside the symbols for some time points).

Hydrogels that can be formed *in situ* under physiological conditions (*i.e.*, *in vivo* gelation) are of interest for diverse biomedical applications, including drug delivery, tissue engineering and regenerative medicine.^15^,^16^ As the results presented herein demonstrate that TiTannic gels are suitable for *in vivo* gelation, we hypothesized that they may be of interest for drug delivery applications. To test this, we compared the *in vitro* drug loading and release properties of TiTannic gels to Pluronic F127 hydrogels. Pluronic F127 gels are currently being explored as an *in situ* gelation system to treat otitis media (ear infection).^17^ In the current study, we loaded Pluronic F127 gels and TiTannic gels with the corticosteroid dexamethasone, and measured drug release using liquid chromatography (Fig. 10). For Pluronic F127 gels, >75% of the drug was released within 3 hours. In contrast, for TiTannic gels >75% of drug was released over 6 days (a >40-fold difference compared to Pluronic F127 gels). Drug release was still at a detectable level (around 1.5–2%) from the TiTannic gel even after 28 days, while for Pluronic F127 no drug release was observed after 2 days. This difference in release rate may be due to the ability of phenols to interact strongly with diverse biomolecules and pharmaceuticals, which may retard the release and enable sustained drug delivery.^42^,^43^,^52^,^53^

## Conclusions

In this study, we demonstrate that metal–phenolic supramolecular gelation can occur successfully *in vivo*, and provide essential *in vivo* characterization for this emerging class of hydrogels. Following characterization with optical and electron microscopy, Raman spectroscopy, and rheological methods—as well as a study of hydrogel porosity and permeability—we assess biocompatibility and immunogenicity following subcutaneous injection in immunocompetent mice. We show that TiTannic gels are stable and well-tolerated, and elicit a mild but persistent foreign body reaction. Through mass spectroscopic analysis of tissue samples, we show that titanium accumulation in distal tissues remains low over the 14 week study period. Finally, we show that TiTannic gels exhibit more sustained release (from <1 day to >10 days) compared to a commonly used *in vivo* gelation system (*i.e.*, Pluronic F127 hydrogels) when loaded with the clinically used corticosteroid dexamethasone. Taken together, these results provide a solid foundation for further exploration of TiTannic gels in biomedical areas such as drug delivery and regenerative medicine.

## Experimental section

### Materials

Tannic acid (TA; Sigma-Aldrich product #403040), titanium(iv) bis(ammonium lactato)dihydroxide solution (Ti-BALDH; Sigma-Aldrich product #388165), sterile dimethyl sulfoxide (DMSO; Sigma-Aldrich product #D2650), sodium hydroxide (NaOH Sigma-Aldrich product #S8045), Pluronic F-127 (Sigma-Aldrich product #P2443), polystyrene microparticles (10% particle dispersion in aqueous solution, 1.1 μm diameter particles; Sigma-Aldrich product #LB11), dexamethasone sodium phosphate (Sigma-Aldrich product #BP108), and 12-well cell culture plates (Sigma-Aldrich product #CLS3513) were purchased from Sigma-Aldrich (USA). The received DMSO was purchased pre-sterilized while other components could be filter-sterilized (0.2 μm syringe filter) after dilution but prior to mixing and usage, if needed. Sterility of DMSO and solutions following sterile-filtration was maintained by working in a biosafety cabinet using standard aseptic procedures.^54^ Millicell cell culture well inserts (0.4 μm pore size, 12 mm diameter; Merck Millipore product #PICM01250) were purchased from Merck Millipore (Ireland). Dulbecco's phosphate-buffered saline (PBS), Dulbecco's modified eagle medium (DMEM), and fetal bovine serum (FBS), were purchased from Thermo Fisher Scientific (USA). Absolute ethanol (>99.8%) was purchased from Honeywell (Germany). Sunflower oil was obtained from a local grocery store (Tesco, UK). Deionized and filtered (0.2 μm pore size) water was used for all experiments (unless otherwise stated) and obtained from a Triple Red water system (Avidity Science, UK). Micropipettes calibrated and certified at least annually were used for all experiments. The MIRIBEL standard^55^ for reporting bio-nano science research and the ARRIVE guidelines^56^,^57^ for reporting research with animals were consulted during the preparation of this manuscript.

### Preparation of TiTannic gels

Full details of the diverse hydrogels and organogels that can be prepared using the TiTannic gel system, including under which conditions and compositions (incl. molar ratios and gelation concentrations) different gelation times can be achieved, has been previously described.^13^ Briefly, a 50 mg mL^–1^ TA solution was prepared in deionized water and the pH was raised to ∼7 using NaOH (1 M, aq.). Separately, DMSO was added to Ti-BALDH until a final concentration of 20 vol%, this Ti-BALDH/DMSO mixture is the Ti^IV^ solution. TA solution and Ti^IV^ solution was mixed through vortexing at a TA : Ti^IV^ molar ratio of around 1 : 2.5 for a gelation time around ∼15 minutes. See Section S1 in the ESI† for additional details including a step-by-step protocol.

### Preparation of Pluronic F127 gels

Pluronic F127 was added to cold PBS (4 °C) to reach a concentration of 18 wt%. This solution was stored in the fridge (4 °C) to keep the solution in a liquid-like stage: at 37 °C the Pluronic F127 solution turns into a gel.^58^,^59^

### Particle tracking analysis

Pluronic F127 and TiTannic gels were prepared as described above to a final volume of 480 μL, which included 10 μL of 10% polystyrene microparticle dispersion which was dispersed in the gel precursor prior to gelation for homogenous distribution throughout the final gel. After gelation for 1 hour at 37 °C, microscopy videos of the particles inside the gels were acquired in bright-field mode using an Olympus BX51 microscope at 10 frames per second for 60 seconds. Particle tracking of individual particles was performed on the acquired videos using the “Particle Tracker” plugin^60^,^61^ of Fiji,^62^ with the output being distance (μm) travelled per individually tracked particle during 60 seconds. The results were plotted using GraphPad Prism 8 (GraphPad Software, USA).

### Rheology

Rheological measurements of the hydrogels were recorded using an Anton Paar MCR302 rheometer fitted with a 25 mm stainless steel parallel plate (PP25) and Peltier temperature controlled hood (P-PTD200/80/I). All measurements were recorded at 298 K, with water added to the Peltier hood to prevent sample evaporation. Time-sweeps were performed with frequency = 10 rad s^–1^, strain = 0.1% and sampling rate = 5 seconds. Amplitude sweeps were performed with frequency = 10 rad s^–1^, log ramp of strain (0.01–100%) and sampling rate = 33 points per decade. Frequency sweeps were performed with strain = 0.1%, log ramp of frequency (0.1–100 rad s^–1^) and sampling rate = 33 points per decade. The results were plotted using OriginPro 2017 and are presented in Fig. S3.†

### TiTannic gel dissolution study

An oil bath using sunflower oil was prepared by pouring the oil into a petri dish until a height from the bottom of the dish to the liquid surface of around 1 cm was obtained. TiTannic gel was prepared as described above. Immediately upon mixing and vortexing TiTannic gel components, 10 μL droplets were manually pipetted directly into the oil bath, resulting in largely spherical aqueous droplets inside the oil bath. These liquid droplets were allowed to gel for 1 hour to form TiTannic gel beads. The gel beads were then transferred into a 50 mL tube of ethanol using disposable transfer pipettes with an opening larger than the bead size. The 50 mL tubes were kept on a tube roller shaker (to ensure continuous mixing) for 1 hour, after which the ethanol was removed and replaced with fresh ethanol. This was repeated three times. During this process the gel beads were washed from oil and sterilized. This washing process was then repeated using sterile PBS (instead of ethanol). After sterilization in ethanol, tubes were only opened inside a biosafety cabinet and handled using aseptic technique and sterilized disposable transfer pipettes. After the final washing step, the beads were added into a sterile petri dish containing PBS which was placed on top of a micrometer ruler and photographs were taken from above the petri dish. The photographs were processed using Fiji,^62^ where the ruler was used to define distance in each photograph which could then be used to measure the size of each bead (∼2 mm in diameter). Following this time point zero measurement, 10 gel beads were added into a 50 mL tube containing 50 mL of PBS, and 10 gel beads were added into a 50 mL tube containing 50 mL of cell culture media (DMEM) supplemented with 10% v/v fetal bovine serum. At pre-determined time intervals, the gel beads were aseptically transferred into a sterile petri dish and the size of each gel bead was again measured following the procedure outlined above. After each measurement, the gel beads were re-dispersed into 50 mL of fresh PBS or fresh DMEM to continue the incubation. During incubation the tubes were kept on a tube roller shaker. After the last time point, the results were plotted using GraphPad Prism 8 (GraphPad Software, USA) and the results are presented in Fig. S7.†

In addition to the study described above, additional gel beads were also added to vials containing PBS, 1 M NaOH, 1 M HCl, or a 0.4 g mL^–1^ aqueous solution of pyrocatechol (Fig. S1b†). Using pyrocatechol to initiate competing ligand-mediated gel disassembly has previously been reported.^14^ Photos of the vials were taken first with all vials containing only PBS (time point = 0 hours) and then at pre-determined time intervals (5 minutes, 1 hour and 24 hours) after replacement of PBS in three of the four vials with NaOH, HCl or pyrocatechol aqueous solutions. Results are presented in Fig. S6.†

### Raman spectroscopy

Raman spectra were obtained using a custom-built Raman spectroscopy system (Fig. S2†) consisting of a multimodal Raman spectroscopy probe with a central excitation fiber surrounded by seven collection fibers (EmVision) connected to a 785 nm diode laser with maximum output of 500 mW (B&W TEK Inc.) for Raman excitation and collected using a fiber-coupled QEPro spectrograph with a 1200 grooves mm^–1^ grating and a 50 μm slit (OceanOptics Inc., Dunedin, FL). The Raman probe has a spot size of around 500 μm and, for comparison, 785 nm light penetration through tissues (depending on the tissue) is on the order of 1 mm.^63^,^64^ Raman spectra were acquired continuously with a 1 second integration time and 100 mW output power using custom, in-house developed scripts (MATLAB 2017a, Mathworks, USA). Spectral processing was also performed in MATLAB and consisted of wavelength calibration using a NeAr lamp, system spectral response correction for CCD dark noise and fiber-optic probe background, fluorescence background subtraction (Whittaker filter, *λ* = 100 000), normalization to the area under the curve, and smoothing using a first-order Savitzky–Golay filter with a frame length of 7.

### Drug loading and release

Three samples of 100 μL Pluronic F127 solution were prepared (each in a 1.7 mL microcentrifuge tube) following the standard protocol described above and kept on ice, with the difference that 60 μg dexamethasone was mixed into each liquid Pluronic F127 sample (final concentration was 60 μg dexamethasone per 100 μL gel). The samples were then gelled for 1 hour by moving these tubes into a heating block kept at 37 °C. 500 μL of PBS (pre-warmed to 37 °C) was then added into each tube to start the elution study.

TiTannic gel was prepared following the standard protocol described above with dexamethasone added to achieve a final concentration of 60 μg dexamethasone per 100 μL TiTannic gel. 100 μL of TiTannic gel mixture was added into each of three 1.7 mL microcentrifuge tubes, this was done immediately upon mixing the TA solution (containing dexamethasone) with the Ti^IV^ solution, before gelation had occurred. The mixtures were then allowed to gel for 1 hour. 500 μL of PBS was then added into each tube to start the elution study. The elution study was also repeated for TiTannic gels but instead of 60 μg dexamethasone per 100 μL gel, 6 mg dexamethasone per 100 μL gel was used to increase the dexamethasone signal per time point to facilitate HPLC detection (as the observed release rate from TiTannic gels were much slower than for the Pluronic F127 gels). In this repeat study, double the amount of Ti^IV^ solution was used as the higher concentration of dexamethasone present affected the gelation time of the TiTannic gel, as assessed with vial inversion testing.

During the elution study, all tubes were incubated in a heating block kept at 37 °C. A sample of fresh PBS (unexposed to the gels) was kept and defined as time point = 0. At each predetermined time point, 500 μL of the PBS was aspirated from each sample and replaced with 500 μL fresh PBS (pre-warmed to 37 °C). Samples were stored at –20 °C until samples from all time points had been collected. Quantification of the dexamethasone concentration of each sample was performed using HPLC, as described below.

### High-performance liquid chromatography (HPLC)

HPLC samples were measured using an Agilent 1260 Infinity Quaternary LC equipped with a Phenomenex Gemini NX C18 column (150 × 4.6 mm, 5 μm pore size and 100 Å particle size). HPLC grade formic acid, water and acetonitrile were used for all analyses and were obtained from Merck. Samples were separated using an isocratic elution of 40% v/v acetonitrile in water with 0.1% v/v formic acid over 10 minutes at a flow rate of 1 mL min^–1^, injection volume of 20 μL, detection wavelength of 240 nm, and a column temperature of 40 °C. The concentration of dexamethasone was determined from a linear calibration curve of the peak areas (Fig. S9†). Samples were analyzed using Agilent OpenLab CDS software to measure peak areas (Fig. S10†). The results were plotted using GraphPad Prism 8 (GraphPad Software, USA).

### Scanning electron microscopy (SEM)

TiTannic gels were prepared (following the standard protocol described above) in 1 mL cut-off syringe tops for easy gel-moulding and removal. Samples were cut from the bulk gels using a biopsy punch and immersed in liquid nitrogen for 1 minute until completely frozen before freeze fracturing using a scalpel. A piece approximately 2 × 2 × 2 mm in size was taken for further processing. The sample was freeze substituted using a Leica EM AFS2 (Leica Microsystems, Germany) by immersion in pure acetone precooled to –90 °C and the sample was ramped to 0 °C at 5 °C per hour. The sample was removed from the acetone and lyophilized, mounted on a SEM stub, and sputter-coated with a 10 nm layer of gold. The sample was imaged using an Auriga Zeiss Crossbeam (Carl Zeiss AG, Germany) in SEM mode at 54° sample tilt (tilt corrected) using the in-lens secondary electron detector and 1.6 kV accelerating voltage. The images were processed using Fiji.^62^

### Permeability assay

A porous well insert (membrane with 0.4 μm pores) was secured in a well of a 12-well cell culture plate filled with stirred 3.5 mL PBS solution. The well insert, containing either no gel (*i.e.*, empty well control), or gel (either 0.2 mL of TiTannic gel or 0.2 mL of Pluronic F127 gel), was positioned so that the permeable bottom was fully immersed in the PBS solution. An in-house fabricated glucose sensor was used to monitor glucose concentration outside of the well insert (Fig. S4†). After a few minutes of stabilization (as monitored by continuous read-out from the glucose sensor), 0.3 mL of either PBS (negative control) or 20 mM glucose solution (in PBS) was added on top of the well insert, *i.e.* on the opposite side of the gel in relation to the glucose sensor. The response of the glucose biosensor was continuously recorded over time with measurements acquired approximately once per second. Evaporation was reduced using a parafilm cover during the assay.

The glucose sensors used in this work are based on and adapted from electrochemical glucose biosensors described elsewhere.^65^–^67^ All monitoring was controlled using in-house potentiostats and a PowerLab 8/35 data acquisition device (ADinstruments, UK), controlled by LabChart Pro (ADInstruments). Data analysis was also performed using LabChart Pro. Current measurements were converted into concentration values using pre- and post-experiment glucose calibrations (by measuring known concentrations of glucose). The results were plotted using GraphPad Prism 8 (GraphPad Software, USA).

### Mice

All animal procedures were performed with UK Home Office approval (UK HO PPL P6F4D9876, holder Dr Susanne Sattler) and conformed to the UK Animals (Scientific Procedures) Act, 1986, incorporating Directive 2010/63/EU of the European Parliament. Healthy, immunocompetent, 8–10 week old, male or female mice of a previously described^68^ hybrid strain (C57Bl/6J × FVB/N × NOD/Shilt) were used. Procedures for the husbandry and housing of animals follow the recommendations of the Association for Assessment and Accreditation of Laboratory Animal Care (AAALAC) and UK Code of Practice for the Housing and Care of Animals Bred, Supplied or Used for Scientific Purposes. Mice were housed in Allentown XJ individually ventilated cages, with ECO2 bedding (Datesand Group, UK) and a 12 : 12 light : dark cycle. RM1 and RM3 chow (Special Diet Service) were provided *ad libitum*. Environmental enrichment was provided including cardboard tubes, wooden chewing blocks, and tissue. The maximum housing density was 5 mice per cage if <25 g and 3 mice per cage if ≥25 g. No animals were involved in previous procedures.

### *In vivo* TiTannic hydrogel injection

100 μL of a sterile preparation of TiTannic hydrogel was injected under aseptic conditions. Mice were anaesthetized using 2% isoflurane and hair was removed from a <1 cm^2^ area on both flanks and treated with Betadine surgical scrub (Fisher Scientific, UK) before injection. TiTannic hydrogels were injected subcutaneously using a sterile 25-gauge hypodermic needle. Mice were maintained under general anesthesia for a total of 10 minutes to allow the gel to form homogenously. Recovery was monitored closely until the mice moved freely and were observed to start feeding again. TiTannic hydrogel with the surrounding skin and subcutaneous adipose tissue layer as well as tissue for Ti detection were isolated after schedule 1 culling of the mice at defined time points.

### Histology

TiTannic hydrogels with surrounding tissues were weighed and measured and fixed in 10% w/v neutral buffered formalin (NBF) overnight, dehydrated in an increasing gradient of ethanol and embedded in paraffin. Five μm sections were cut, de-waxed and rehydrated in an ethanol gradient. Sections were stained with haematoxylin and eosin (H&E) and Picrosirius Red/Fast Green. All reagents were purchased from Sigma-Aldrich. Sections were also stained for the pan-leukocyte marker CD45 using anti-mouse CD45 antibody clone: 30-F11 (BioLegend, UK) and detection using HRP-labelled goat anti-rat IgG (Vector Laboratories, USA). The DAB substrate kit (Vector Laboratories, UK) was used according to manufacturer's instructions. After final dehydration, slides were mounted with DPX mountant (Sigma-Aldrich) and analyzed using a Nikon Eclipse TE2000 inverted microscope or a LMD7000 microscope (Leica microsystems, UK). Measurements were obtained using Fiji^62^ and included the total area of the gel, the thickness of the layer of infiltrate and fibrosis surrounding the gel, and the thickness of the material/cell overlap layer. The number of foreign body giant cells were counted manually as shown in Fig. S14.†

### Titanium measurements and biodistribution

Titanium was measured in blood, urine and tissue samples with a Thermo Element 2 magnetic sector field HR-ICP-MS (Thermo Fisher Scientific, Germany). Calibration standards were prepared by dilution from a custom stock solution (QMX Laboratories Limited, UK) with a titanium concentration traceable to NIST SRM 3162a Lot 130925. Separate matrix-matched calibrations were prepared for each sample type to minimize matrix effects.

150 μL samples of blood or urine were mixed with 150 μL of water and 4.5 mL of assay diluent: 0.5% (v/v) tetramethylammonium hydroxide (electronics grade, Alpha Aesar, US), 0.005% (v/v) Triton X-100 (Romil, UK) and 2.5 μg L^–1^ gallium (Alpha Aesar, US). Blood and urine calibrators comprised 150 μL of each standard with 4.5 mL of assay diluent and 150 μL of either defibrinated horse blood (TSC Biosciences, Buckingham, UK) or deionized water (urine calibrators and blank).

Tissue samples up to 150 mg in weight were accurately weighed (Sartorius CP124S analytical balance) and made up to 300 mg with deionized water. Tissue calibrators comprised 150 μL of each calibration standard and either 150 μL defibrinated horse blood (TSC Biosciences, UK) or deionized water (blank). 1 mL of 25% (w/w) aqueous tetramethylammonium hydroxide (electronics grade, Alpha Aesar, US) was added to the tissue samples and calibrators and incubated for 48 hours at room temperature. 1% (v/v) nitric acid containing 5 μg L^–1^ gallium (Alpha Aesar, US) was added to partially neutralize the sample prior to analysis.

The diluted samples and calibration standards were sequentially sampled using an ESI-SC FAST autosampler (Elemental Scientific, US) and introduced to the HR-ICP-MS with a PTFE Nebulizer (Elemental Scientific, US) and cyclonic spray chamber (Thermo Scientific). Ti^47^ and Ga^71^ were measured in medium resolution mode. The counts per second (cps) data for Ti^47^ cps were normalized to Ga^71^ cps and calibration curves plotted in Microsoft Excel.

During the period of study, the laboratory was enrolled in the Quebec Multielement External Quality Assessment Scheme for blood titanium measurement and submitted results that were close to target and well within the acceptable range.

## Author contributions

M. B. developed the overall idea and experimental design, conducted experiments and analyzed the data, prepared the first draft of the manuscript, and led the project. L. M. W. contributed to the animal research studies. J. P. W. contributed to the drug elution studies and to the rheology studies. J. P. contributed to the electron microscopy studies. C. C. H. contributed to the Raman spectroscopy studies. M. A. B. contributed to the glucose permeability assays. N. G. M. contributed to the mass spectroscopy studies. S. S. led the animal research studies in the project and contributed to overall experimental design and data analysis. M. M. S. supervised the project. All authors contributed to the preparation of the manuscript and approved its submission for publication.

## Conflicts of interest

The authors declare no competing interests.

## Supplementary Material

Supplementary information
