# Supplementary material for: In vivo biocompatibility and immunogenicity of metal–phenolic gelation
Source: Chem Sci. 2019 Sep 25;10(43):10179–94. doi: 10.1039/c9sc03325d (PMC6837883; doi:10.1039/c9sc03325d)
Supplement: Supplementary file 1 [file SC-010-C9SC03325D-s001.pdf]

Electronic Supplementary Material (ESI) for Chemical Science.
This journal is © The Royal Society of Chemistry 2019

SUPPORTING INFORMATION

In vivo biocompatibility and immunogenicity of metal-phenolic gelation

Mattias Björnmalm,[1] Lok Man Wong,[2] Jonathan P. Wojciechowski,[1] Jelle Penders,[1] Conor C. Horgan,[1] Marsilea A. Booth,[1] Nicholas G. Martin,[3] Susanne Sattler,[2] Molly M. Stevens[1]**

Affiliations

[1]Department of Materials, Department of Bioengineering, and Institute of Biomedical Engineering, Imperial College London, London SW7 2AZ, UK

[2]National Heart and Lung Institute, Imperial College London, London W12 0NN, UK

[3]Trace Element Laboratory, North West London Pathology, Charing Cross Hospital, London W6 8RF, UK.

*Corresponding authors: s.sattler@imperial.ac.uk (S.S.), m.stevens@imperial.ac.uk (M.M.S.)

ORCIDs

M.B.: https://orcid.org/0000-0002-9876-7079

L.M.W.: https://orcid.org/0000-0002-8559-0606

J.P.W.: https://orcid.org/0000-0002-6272-515X

J.P.: https://orcid.org/0000-0002-5232-917X

C.C.H.: https://orcid.org/0000-0003-0495-1615

M.A.B.: https://orcid.org/0000-0001-9778-905X

N.M.: n/a

S.S.: https://orcid.org/0000-0001-9932-4109

M.M.S.: https://orcid.org/0000-0002-7335-266X

Section 1. A step-by-step protocol for preparation of TiTannic gels

Materials

- Tannic acid (TA; Sigma-Aldrich product #403040)
- Titanium(IV) bis(ammonium lactato)dihydroxide solution (Ti-BALDH; Sigma-Aldrich product #388165)
- Sterile dimethyl sulfoxide (DMSO; Sigma-Aldrich product #D2650)
- Sodium hydroxide (NaOH Sigma-Aldrich product #S8045), dissolved in deionized water to 1 M. Sterile filter.
- Use 1.7 mL microcentrifuge tubes (pre-sterilized by autoclaving).

Method

Preparation

1. Weigh 200 mg TA.
2. Add into 4 mL deionized water in 50 mL tube. Vortex and then leave on tube rotator until fully dissolved (ca. 15 minutes). The result is 5 wt.%, 50 mg mL^{-1} TA solution.
   a. Can be sterile filtered with a 0.2 μm syringe filter if needed.
   b. Note: TA solution is ideally prepared fresh as it may oxidize over time.
3. In new 1.7 mL tube: mix 400 μL Ti-BALDH (sterile filter first if needed) with 100 μL DMSO. This mixture is the Ti^{IV} solution.
   a. Note: If sterility is needed, perform this step in a biosafety cabinet using aseptic technique.

Make gel

4. In a new 1.7 mL tube: Mix 400 μL TA solution with 50 μL NaOH (1 M, aq.). This raises pH to ~7.
5. Add Ti^{IV}, vortex to mix and let stand to gel.
   a. For 1:5 molar ratio (TA:Ti) as described in the original publication[1]: add 43.2 μL Ti^{IV} solution. Gels very quickly (<1 minute).
   b. Unless otherwise stated, 17 μL of Ti^{IV} solution was used in the current study resulting in a ~15 minutes gelation time.
      i. Note: If knowing the exact gelation time is important (e.g. for animal experiments) it is recommended to test the gelation time each time after components have been freshly prepared. This can be done by simple inversion testing (see Figure 1a). For example, to test for ~15 minutes gelation time, add and vortex a few different amounts of Ti^{IV} solutions: e.g., 14 μL, 17 μL, and 20 μL of Ti^{IV} to different tubes of 450 μL TA solution (with NaOH already added) prepared as per step 4 above and measure the time until gelation.
   c. Note: pH can be measured with a pH meter and/or with a pH indicator strip to ensure physiological pH before usage. NaOH (as above) can be used to further adjust pH if needed.
   d. Note: Many compositions (with diverse gelation times) of TiTannic gels have previously been reported. For examples, see the original publication and its supporting information.[1]

Section S2. Supporting figures

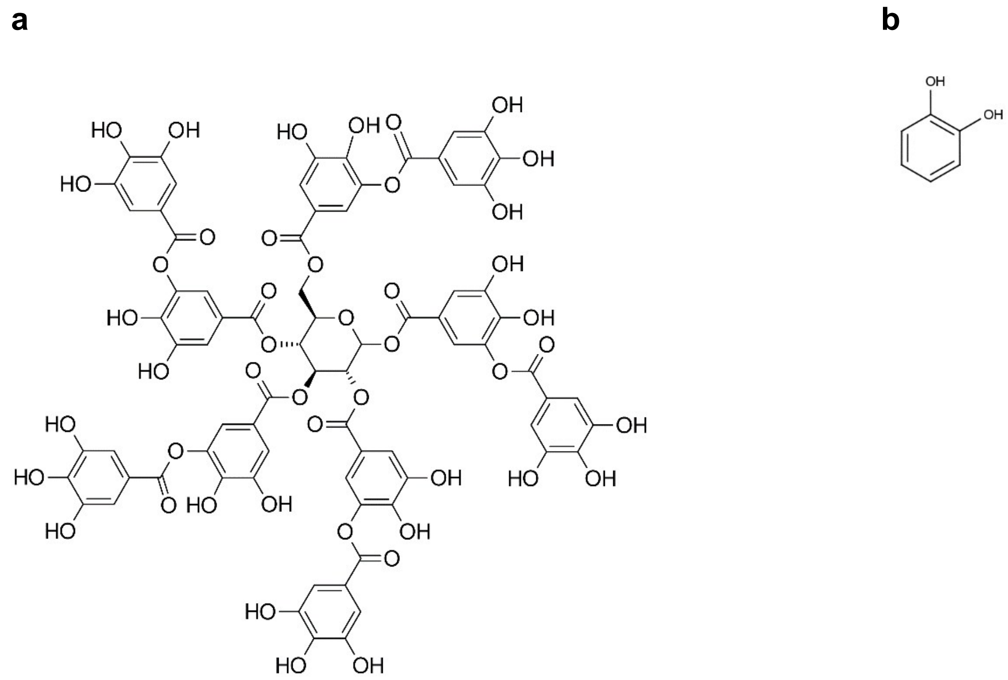

Figure S1. (**a**) Idealized molecular structure of tannic acid with a molecular mass of 1701.2 g mol^{-1}. Note that this is an idealized structure of tannic acid and that the length of each poly(gallic acid) arm can vary (which can then also affect the overall molecular weight). (**b**) Molecular structure of pyrocatechol. Molecular mass is 110.1 g mol^{-1}.

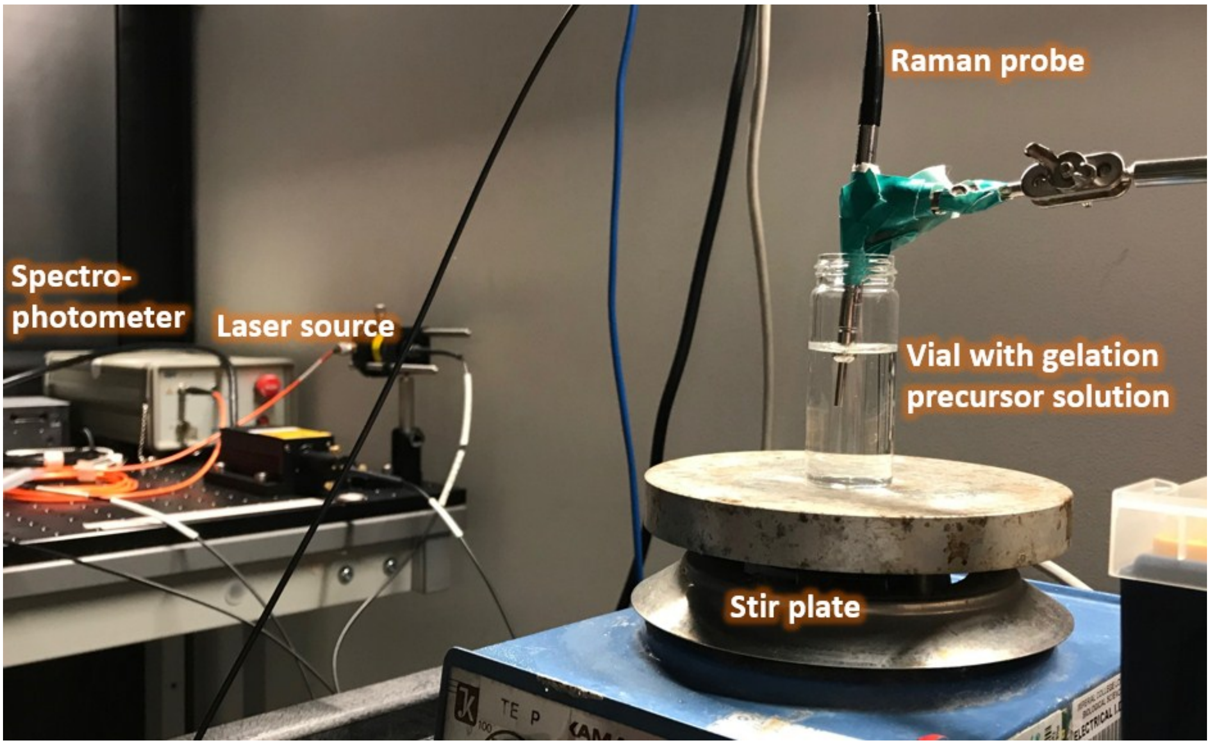

Figure S2. Annotated photograph of the *in situ* Raman spectroscopy probe set-up. The probe is immersed in TA solution (and remains immersed during the experiment) and set to continuously acquire spectra (1 second integration time). When Ti^{IV} solution is added then this is defined as time = 0. A magnetic stir bar and stir plate is used to thoroughly mix the solution for 10 seconds and is subsequently turned off.

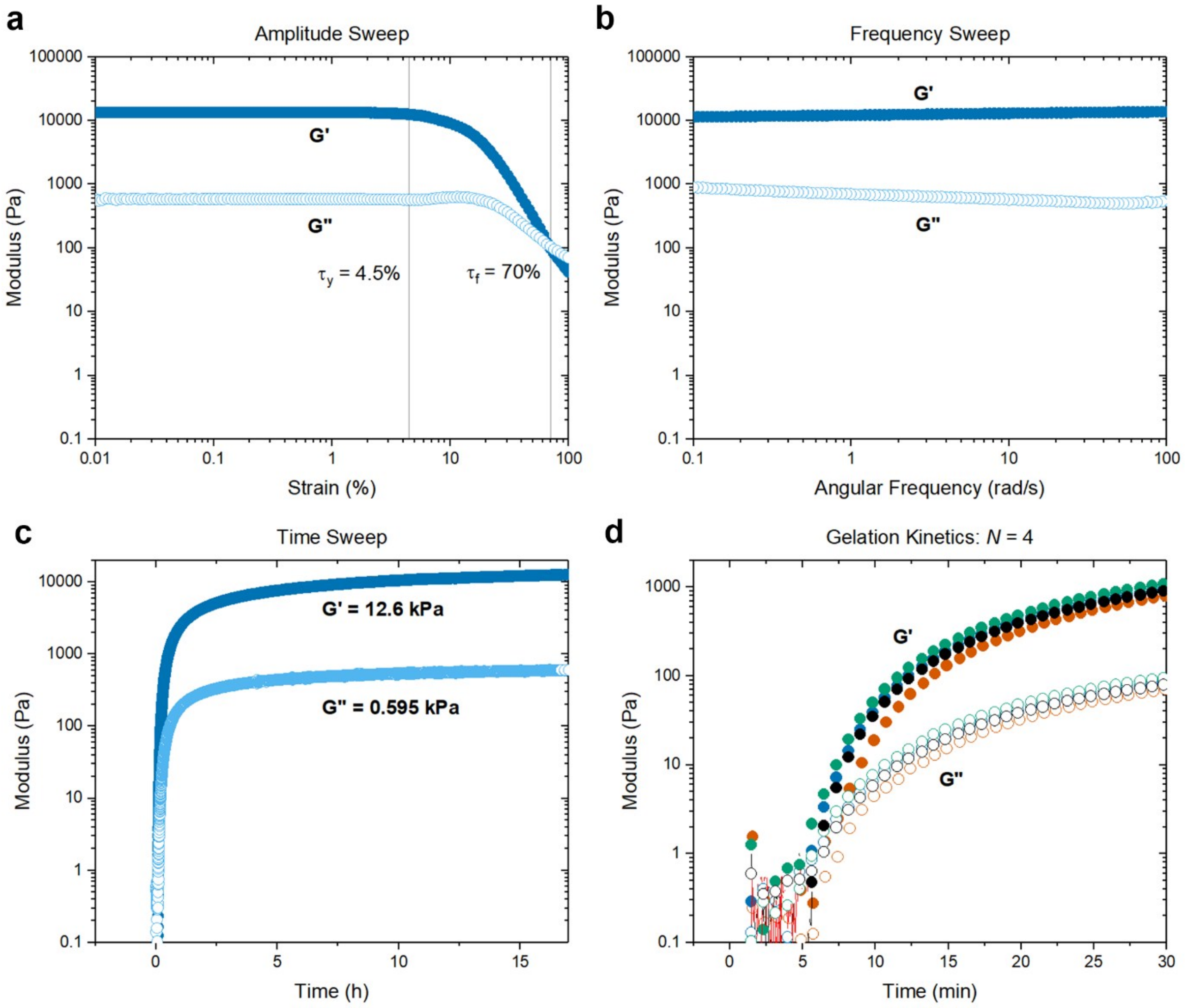

Figure S3. Rheological data for the TiTannic hydrogels. (**a**) Amplitude sweep for the TiTannic hydrogels showing a linear viscoelastic region up to τ_y = 4.5% and a crossover point at τ_f = 70%. (**b**) Frequency sweep showing frequency-independent behavior of the TiTannic gels. (**c**) Time sweep for the TiTannic hydrogels showing a plateau storage modulus G′ = 12.6 kPa and a plateau loss modulus G″ = 0.595 kPa. (**d**) Gelation kinetics (N = 4) within the first 30 minutes for the TiTannic hydrogels.

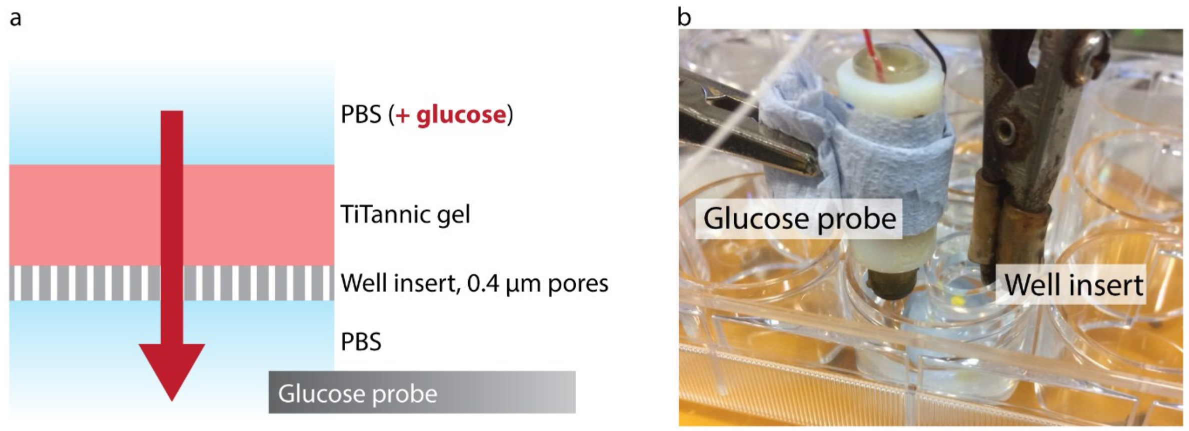

Figure S4. Schematic (**a**) and annotated photograph (**b**) of glucose probe set-up. A gel is cast inside the well insert to form on top of the permeable bottom. The glucose probe is immersed into PBS inside the multi-well plate and the well insert is added adjacent to the glucose probe. The glucose probe and the well insert are held in place using "crocodile clips", but these are not in contact with the sample solutions. PBS containing glucose is added into the well insert and any glucose that diffuses through the gel and the permeable membrane at the bottom of the well insert can reach the glucose probe in the well of the multi-well plate. In this fashion glucose diffusion and permeability through the gel can be monitored.

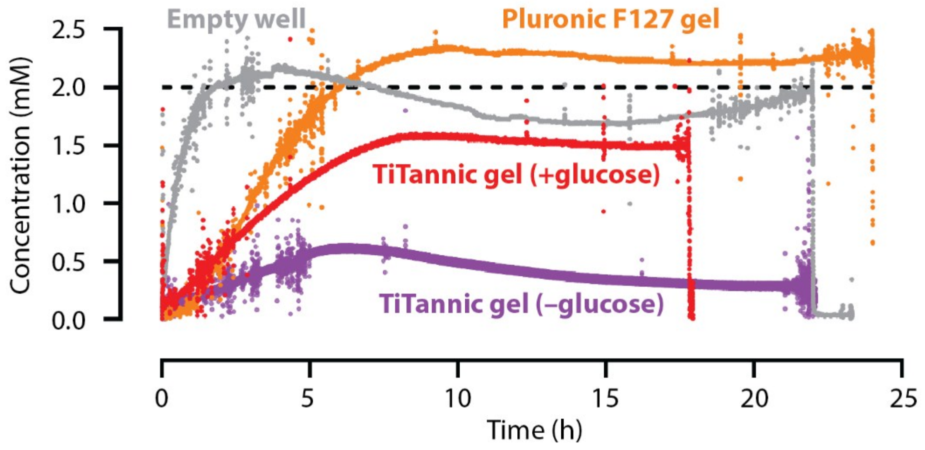

Figure S5. Measured glucose permeability for a TiTannic gel and a Pluronic F127 gel. An empty well (no gel added) was used as a control, representing free diffusion of glucose through the well insert. The dotted black line corresponds to 2.0 mM glucose concentration which is the equilibrated glucose concentration on both sides of the well insert membrane

(i.e. full permeability). TiTannic gel without any glucose added was used as a control to confirm that the gel itself did not induce any substantial signal with the glucose probe.

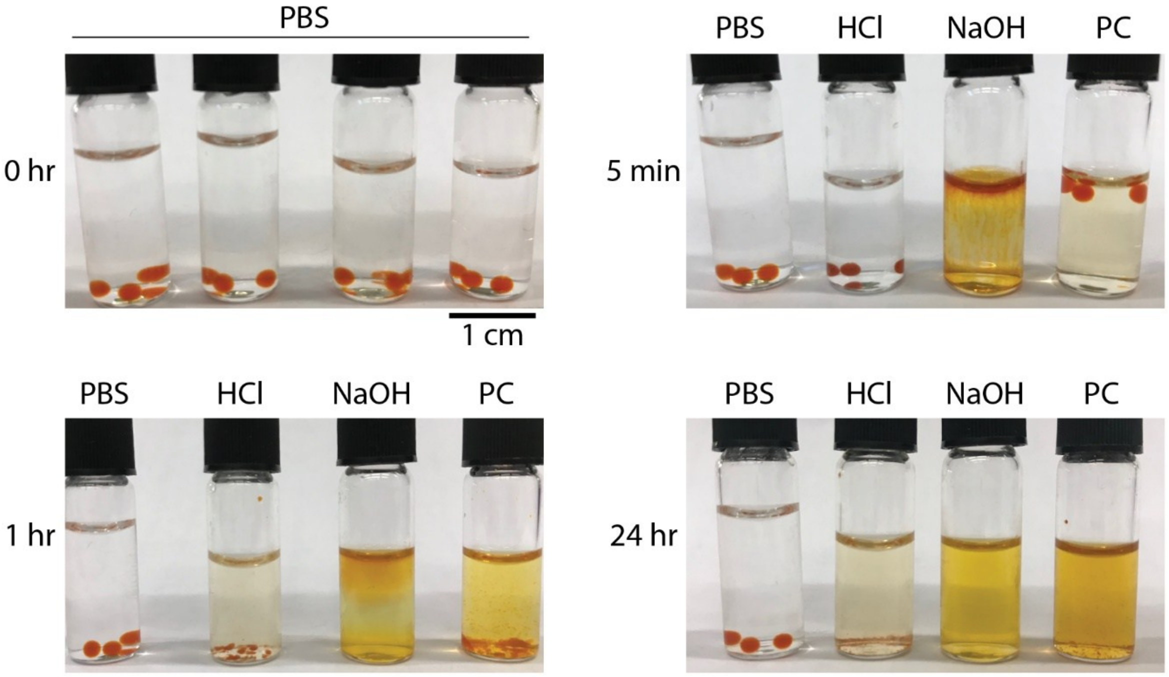

Figure S6. TiTannic gel beads (~2 mm diameter) disassemble under extreme pH (1 M NaOH or 1 M HCl) or in the presence of competing ligand pyrocatechol (PC; Figure S1b). Beads submerged in NaOH are largely disassembled within the first few minutes, while beads submerged in HCl or PC disassemble within a few hours. After 24 hours, all beads except for those immersed in PBS have disassembled. Gel beads immersed in PBS or cell culture media are largely stable over several months (Figure S7).

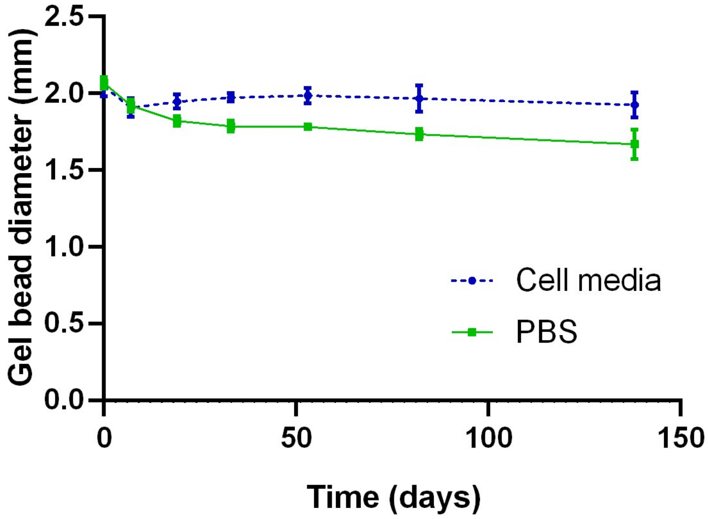

Figure S7. TiTannic gel beads (~2 mm diameter) are largely stable in sterile DMEM cell culture media and PBS over several months. Similar behavior is observed for alginate beads under sterile *in vitro* conditions.[2] The diameter of each gel bead was measured with ImageJ software using calibrated micrographs taken at each time point. No substantial swelling or shrinkage of the gels was observed, which is in agreement with the in vivo results (Figure 5). Gel beads can disassemble rapidly under low or high pH or in the presence of a competing ligand (Figure S6).

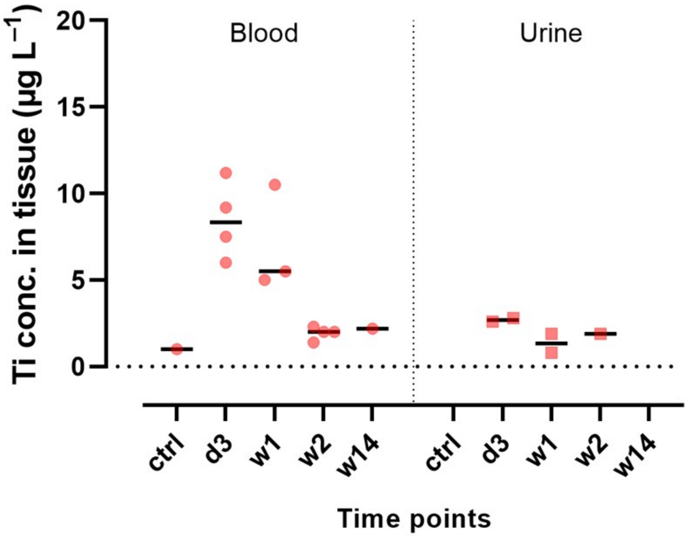

Figure S8. Blood titanium levels increase at early time points following subcutaneous injection of TiTannic gel, while urine levels remain low. Quantification of titanium levels

was performed using mass spectroscopy. Control samples (ctrl) are from animals that were not exposed to the TiTannic gel. Tissues from animals exposed to TiTannic gels were collected 3 days (d3) and 1, 2 and 14 weeks (w1, w2, w14, respectively) following subcutaneous injection of TiTannic gel. For ‘blood ctrl’, ‘blood w14’ and ‘urine w2’ samples were pooled to obtain sufficient material for analysis. For ‘urine ctrl’ and ‘urine w14’ insufficient material was obtained to conduct the analysis. Data points indicate individual values (N = 1–4), and horizontal lines median value.

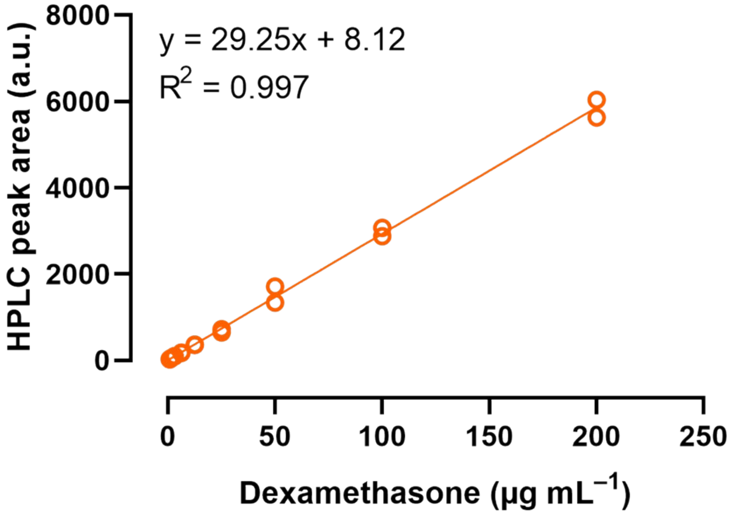

Figure S9. HPLC standard curve for dexamethasone. Data points are measurements from the individually prepared duplicate dilution series. The line is a linear regression curve, and the inset shows the equation for this curve with the calculated goodness of fit (R^2 value).

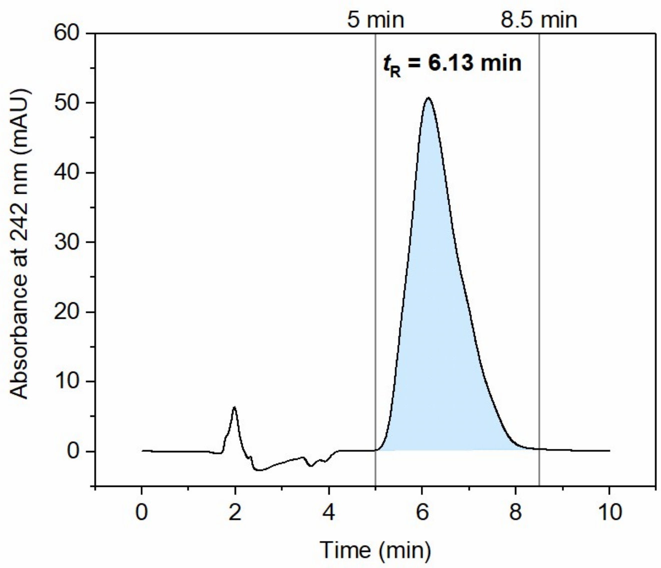

Figure S10. Example of integration settings used to determine area-under-curve of HPLC signal. Representative HPLC trace of dexamethasone-phosphate obtained for release studies. Area under the curve used to determine concentration is highlighted in blue, measured from 5–8.5 minutes. ChemStation software integration settings: slope sensitivity = 1; peak width = 0.02; area reject = 1; height reject = 1.7; shoulders = OFF.

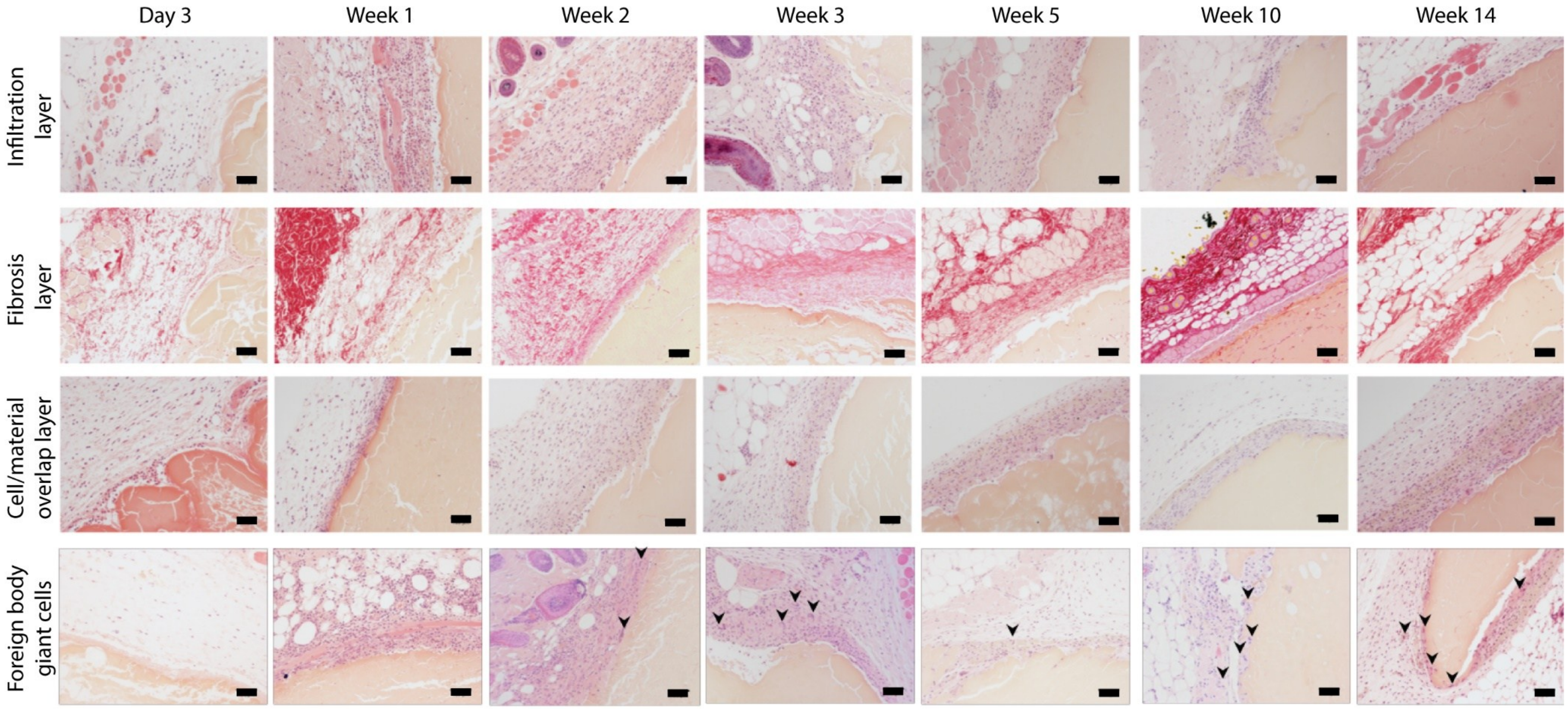

Figure S11. Examples of haematoxylin and eosin staining (infiltration, cell/material overlap, foreign body giant cells) and Picrosirius red (fibrosis) histology micrographs over time. In the FBGC row, an asterisk indicates macrophage-derived FBGC and arrow indicates Langerhans cell-derived FBGC. Scale bars are 100 µm.

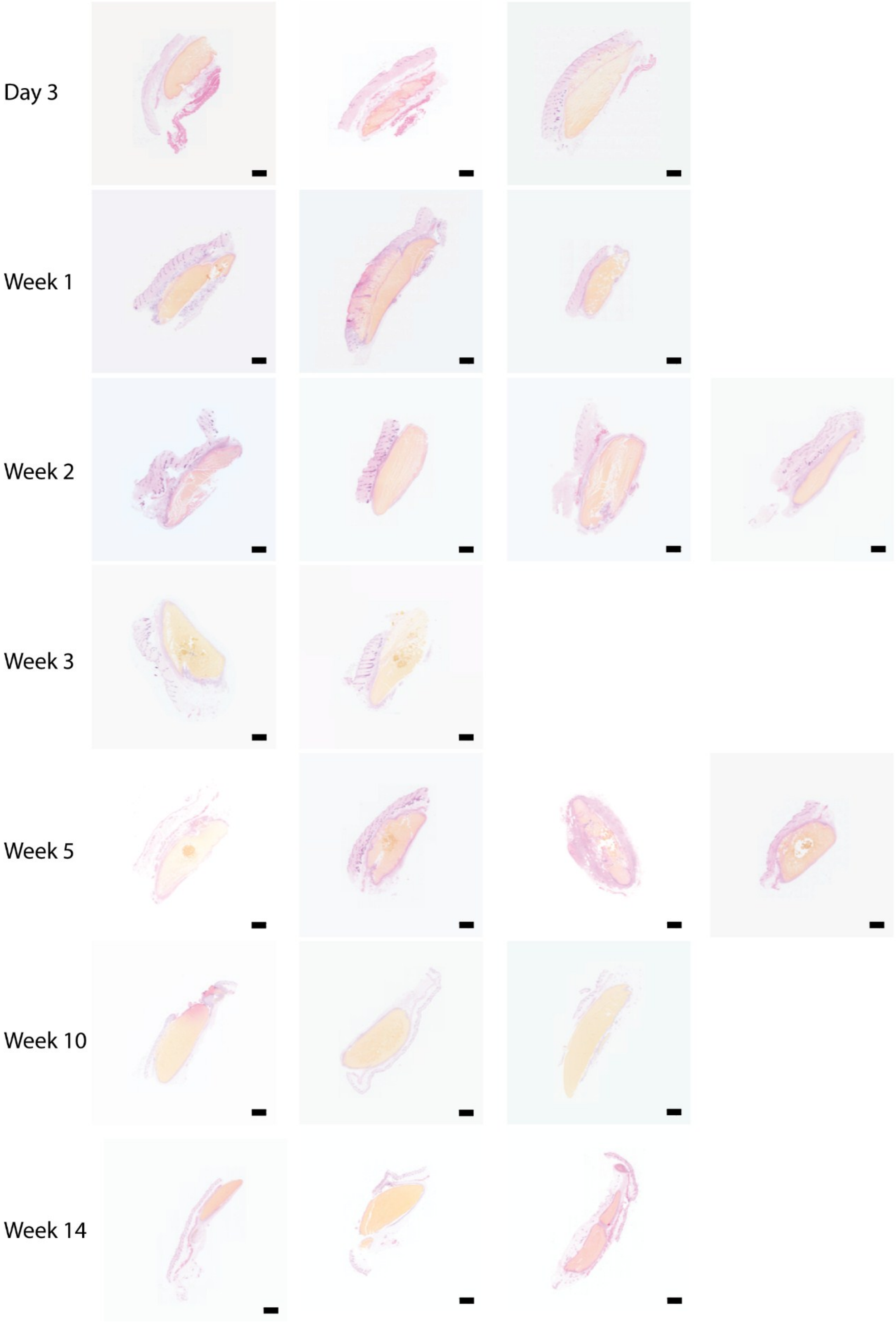

Figure S12. Histology of TiTannic gels. Haematoxylin and eosin (H&E) histology staining of paraffin-embedded TiTannic gel cross-sections over time (H&E; blue nuclei, pink cytoplasm, yellow TiTannic gel). Scale bars are 1000 μm.

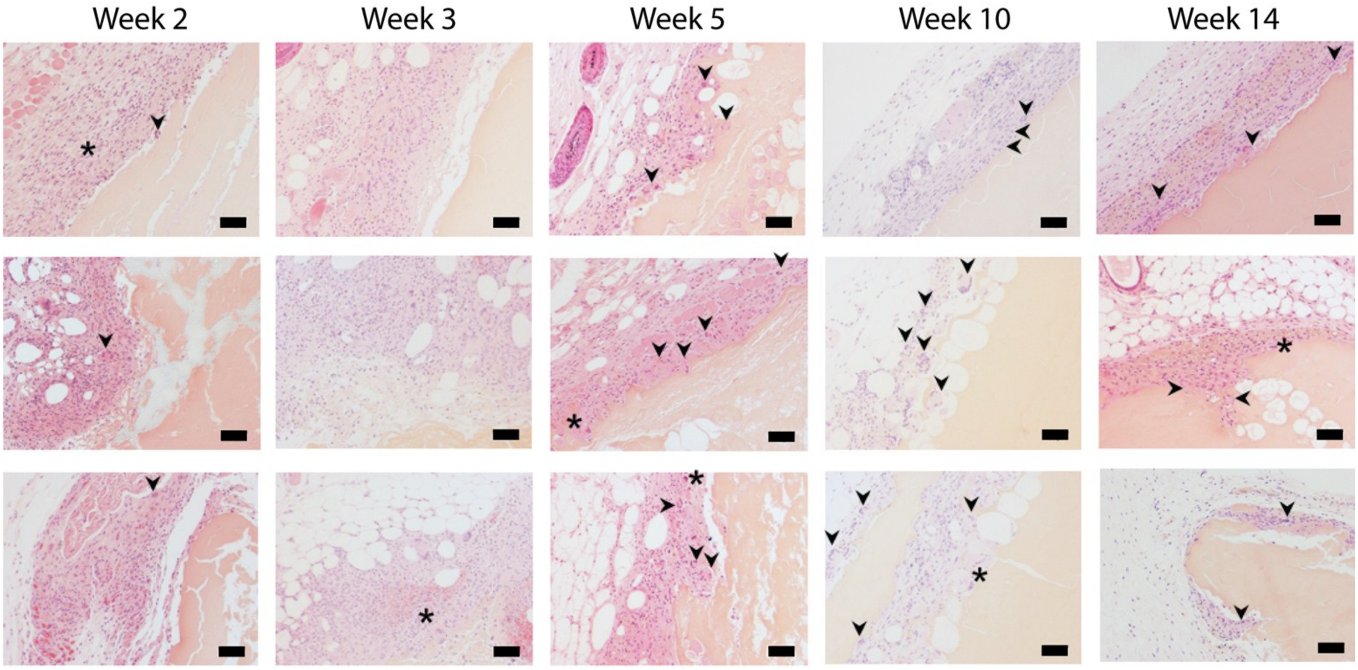

Figure S13. Foreign body giant cells. Three representative micrographs per time point of H&E-stained histology sections showing classical foreign body giant cells (asterisk) and Langerhans giant cells (arrow) in response to TiTannic gel over time. Scale bars are 100 µm.

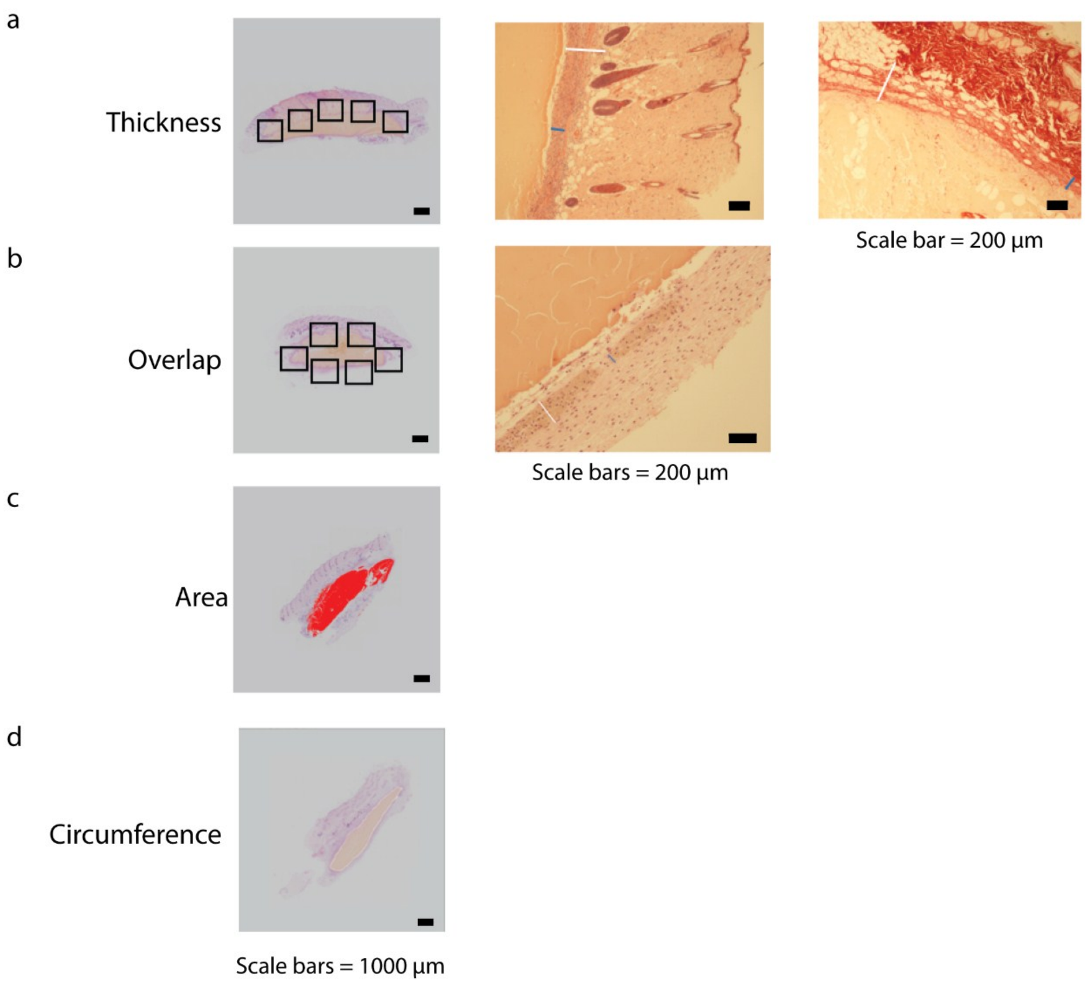

Figure S14. FIJI measurements for quantification of (**a**) thickness of infiltration and fibrotic layer (thickness = Σ / 10), (**b**) cell/material overlap layer (thickness = Σ / 12), (**c**) gel cross sectional area and (**d**) foreign body giant cells (FBGC) per gel circumference (FBGC = Σ / circumference). Thickness measures were taken as two measurements in each field of view representing the shortest (blue) and widest (white) distance. Scale bars in the leftmost column are 1000 µm, and the other scale bars are 200 µm.

Section S3. References

1. Rahim, M. A.; Björnmalm, M.; Suma, T.; Faria, M.; Ju, Y.; Kempe, K.; Mullner, M.; Ejima, H.; Stickland, A. D.; Caruso, F., Metal-phenolic supramolecular gelation. *Angew. Chem. Int. Ed.* **2016,** *55*, 13803-13807.
2. Moya, M. L.; Morley, M.; Khanna, O.; Opara, E. C.; Brey, E. M., Stability of alginate microbead properties in vitro. *J. Mater. Sci. Mater. Med.* **2012,** *23*, 903-912.
